# Safety of Families

**Published:** 1877-10

**Authors:** 


### HALL'S JOURNAL OF HEALTH.

Our Legitimate Scope is almost boundless: for whatever begets pleasurable and harmless feelings, promotes Health; and whatever induces disagreeable sensations, engenders Disease.

WE AIM TO SHOW HOW DISEASE MAY BE AVOIDED, AND THAT IT IS BEST, WHEN SICKNESS COMES, TO TAKE NO MEDICINE WITHOUT CONSULTING A PHYSICIAN.

Vol. 24.] OCTOBER, 1877. [No. 10

### SAFETY OF FAMILIES.

One of the very best means for preserving the health, happiness, and morals of sons and daughters, for raising them up to occupy high, responsible, and honorable positions in society, and for securing to them an old age of quiet repose, with a happy freedom from wasting and wearing diseases of mind and body, is to make home, the family fireside, the companionship of parents and one another, the sweetest, happiest, and most delightful place of all others. Taking into consideration the intensely inquiring character of the youthful mind, and the tendency in all to regard as true what is put in print, there is, perhaps, no other one method of bringing up a loving and lovable family, of securing a happy household, than that of supplying the children with suitable reading from the time they are first able to read at all. There may be some difference or opinion as to what kind of reading is most suitable, but the great mass of the intelligent and the good will have no difficulty in arriving at the conclusion that in the main it should be such as will combine truthfulness with interest. Fill and feed the mind with facts in language which shall engage the attention; facts, and truths, and histories which lead out the affections, the best feelings of the human heart, which will wake up the sympathies to a healthful and practical exercise. There is no scene in domestic life so purely beautiful, except that of family worship, than that of father, mother, children, all gathered around the table, before a cheerful, blazing fire, of a winter's evening, reading aloud by turns, with intervals of remark as to the sentiments conveyed, their application to the times or to one another, their literal correctness, the propriety of the modes of expression, and the many other points which may be suggested to the mind of reader or listener, as page after page is passed over. Very many articles might be selected from different writers as an example of the miscellaneous reading which might, with advantage, come before a family once a month. The subjects are various enough and practical enough, and withal truthful enough to engage the attention, impart instruction, and lead out the mind to thoughtful inquiry and to practical action in any family circle which might meet together. All the articles are truthful. Fact and not fiction is the best nourishment, the most appropriate food for young minds; to feed them on imaginary narrations is as inevitably pernicious to the mind as the habitual use of stimulants is to the body. An early grave, or a life of poverty, dishonor, and bodily suffering, is the fate of those who "drink;" just as certainly will those who feed daily on fiction "spoil" the mind, weaken it, unfit it for the duties of life, and for the high and holy exercise of the sympathies and the best feelings of our nature. Novel-reading is the parent of selfishness, of hard-heartedness, and of a wayward, aimless, fruitless life. The last persons in the world to devote themselves to the beneficence of life, to the practical charities which so elevate us, are novel-writers and novel-readers. The blessings of the good be upon him who, reading this article, shall resolve that there shall be at least one family magazine in the world which shall come every month to eager households, freighted with all that is beautiful in sentiment, truthful in narration, and in matter instructive, pure, and elevating, to be read aloud in the family; of the advantages of which a recent writer well says:

"Books and periodicals should be angels in every household. They are urns to bring us the golden fruits of thought and experience from other minds and other lands. As the fruits of the trees of the earth's soil are most enjoyed around the family board, so should those that mature upon mental and moral boughs be gathered around by the entire household. No home exercise could be more appropriate and pleasing than for one member to read aloud for the benefit of all. An author's ideas are energized by the confidence and love of the tender family affections, and every heart is open to the truth, like the unfolded rose, to receive the gathering dews. The ties of love between parents and children, and brothers and sisters, are thus cemented yet more and more, and varied charms and pleasures are constantly open through this medium to make a home a very paradise. If parents would introduce this exercise in their families, they would soon see the levity and giddiness that make up the conversation of too many circles giving way to refinement and chaste dignity. Read to your children, and encourage them to read to you, instead of reading your papers and books in silence, and in silence laying them away." Thus making home inviting, cheerful, and happy, the sons will be kept from the contaminating influence of the street, the corner-grocery, the engine-house, and the tavern, while the daughters will grow up loving, domestic, virtuous, and pure, and both sons and daughters will live happily, healthfully, usefully, and long.

